# Economic evaluation of monoclonal antibody in the management of colorectal cancer in Malaysia

**DOI:** 10.1186/1472-6963-12-S1-P3

**Published:** 2012-11-21

**Authors:** MS Natrah, Sharifa Ezat WP, MA Syed, Mohd AM Rizal, S Saperi, S Ismail, I Fuad, Muhd MA Azrif

**Affiliations:** 1 National University of Malaysia, Cheras, Kuala Lumpur, Malaysia; 2 United Nations University International Institute for Global Health, Kuala Lumpur, Malaysia

## Introduction

The introduction of monoclonal antibody in the management of Colorectal Cancer (CRC) in the rapidly rising healthcare cost environment prompt a proper evaluation of its cost effectiveness especially to the local setting. The rising incidence of CRC in Malaysia also justifies a detailed evaluation on its economic impact. This study aims to determine the cost of CRC management and to compare the cost effectiveness of monoclonal antibody with conventional chemotherapy in the management of CRC.

## Methods

This economic evaluation study was performed from the societal perspective. It involves collecting resource utilization data based on clinical pathway of colorectal cancer management. Cost calculated included cost of drugs, human resources, administrative, investigations as well as capital cost. Direct and indirect patient’s cost were also calculated based on interview with CRC patients. CRC patients’ quality of life were measured using EORTC QLQ-C30 questionnaire and effectiveness estimates for monoclonal antibody (Cetuximab and Bevacizumab) treatment were modeled from study respondents based on references from other studies. One way sensitivity analysis was used to determine the robustness of the result.

## Result

A total of 160 respondents were involved in the study with the mean age of 58.47 (SD 12.04) years. The average cost of treating a case of colorectal cancer is RM 22,833.44 (RM 18,818.53 - RM 26,848.35). Cost of CRC management increased with increasing stage of the disease (Kruskal Wallis, X^2^ = 106, p < 0.001). Incremental cost per life years gained is RM 118,366.37 for Cetuximab and RM 61,584.68 for Bevacizumab. Incremental cost per quality adjusted life years gained for Cetuximab is RM 67,063.83 and RM 34,892.47 for Bevacizumab. Although both types of monoclonal antibody are considered cost effective (based on WHO guideline of less than 3 times of GDP), Bevacizumab is considered more cost effective than Cetuximab. Sensitivity analysis shows that, cost effectiveness was sensitive to the percentage of late stage of CRC.

## Conclusion

Monoclonal antibody especially Bevacizumab is more cost effective in the management of late stage of CRC. Therefore it should be considered to be used in the CRC as a combination with the current chemotherapy treatment for CRC. The country should invest to the administration of monoclonal antibody to CRC treatment.

